# Calcaneal fractures: Where are we now?

**DOI:** 10.1007/s11751-017-0297-3

**Published:** 2017-10-20

**Authors:** Aisha Razik, Mark Harris, Alex Trompeter

**Affiliations:** St George’s Hospital, Blackshaw Road, Tooting, London, SW17 0QT UK

**Keywords:** Calcaneal fractures, Soft tissue, Intra-articular, Comminution, Operative techniques

## Abstract

This review article on the current management for calcaneal fractures discusses the advantages and disadvantages of different treatment options including the problems encountered. Controversies are described and the evidence reviewed. The management of some types of displaced intra-articular calcaneal fractures remains contentious; is there a preferred stabilisation method for each type of calcaneal fracture? How constant is the “constant fragment” in an intra-articular calcaneal fracture and what is the evidence for primary arthrodesis and what is its place in these fractures?

## Introduction

### Epidemiology

Calcaneal fractures account for up to 75% of all foot fractures and 1–2% of all fractures [1], being more common in males and those who work in an industrial profession. This has socio-economic consequences; male labourers who sustain bilateral intra-articular fractures and have support from compensation benefit carry a poorer prognosis [2].

### Anatomy

The applied surgical anatomy of the calcaneus is complex due to the multifaceted nature of the bone and its articulations with the cuboid and the talus. The calcaneus is made up of a superior articular surface comprising three articulating facets—a posterior, middle and anterior. The sustentaculum tali is a medial bony projection supporting the neck of the talus. The sinus tarsi is a calcaneal groove comprising the anterior and middle facet and the talar sulcus. Benirschke et al. [3] discuss anatomical features relevant to the understanding of managing calcaneal fractures. The lateral side of the calcaneus and its flat nature is highlighted as the most advantageous for internal fixation, but the poor soft tissue cover challenges wound healing. The medial wall is associated closely with the posterior tibial neurovascular bundle and its branches making the surgical approach challenging. The sustentaculum tali is thought to be the most stable part of the calcaneus and relies on supporting tendons in maintaining its anatomical position in most fractures [3–5].

The blood supply to the medial side of the calcaneus is from perforating branches from the posterior tibial artery. The lateral calcaneal artery, which can be a branch of the posterior tibial artery or the peroneal artery, supplies most of the lateral side. Ten per cent of the blood supply has been found from a cadaveric study to come from the sinus tarsi artery. Within the bone, there is a watershed area where the medial and lateral intra-osseous arteries anastomose in the midline [6].

### Mechanism of injury

Most calcaneal fractures are caused by axial loads, e.g. a fall from a height. Primary and secondary fracture lines develop. The primary fracture lines run through the posterior facet of the subtalar joint creating a superolateral fragment and a superomedial or “constant fragment” which includes the sustentaculum tali. If this force continues even further, a secondary fracture line is created and depending on the direction of the force, a tongue-type fracture or joint depression-type fracture will form.

Essex-Lopresti suggested if the secondary fracture line propagated back to the posterior border of the tuberosity, a tongue type occurred [7]. Conversely, if the force was behind the joint but across the body and out between the posterior facet and the level of the insertion of the tendo Achilles, then the more common joint depression-type fracture occurred. If the tuberosity is forced upwards with displacement and the primary fracture line cleaves open, this becomes a severely comminuted fracture with a potential compromise to the blood supply and avascular necrosis [7]. Tongue-type fractures have been associated with posterior skin breakdown, and severely displaced tongue-type fractures should be considered a surgical emergency [8].

Extra-articular calcaneal fractures make up approximately 25% and are caused usually by an avulsion to the anterior process, the sustentaculum tali or the calcaneal tuberosity. Intra-articular calcaneal fractures are the majority. Essex-Lopresti described two groups—those not involving the subtalar joint (25%) and those which do, and it is this which is most challenging technically and has a worse prognosis [9].

### Classification of intra-articular fractures

**Fig. 1:**
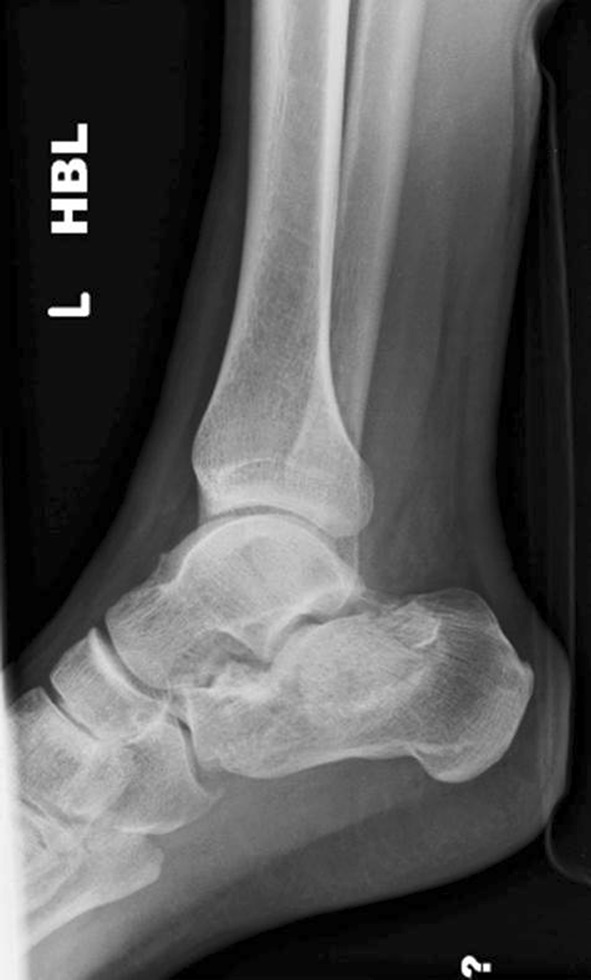
A 30-year-old male with a joint depression-type fracture. The heel is short, in varus and laterally translated

**Fig. 2:**
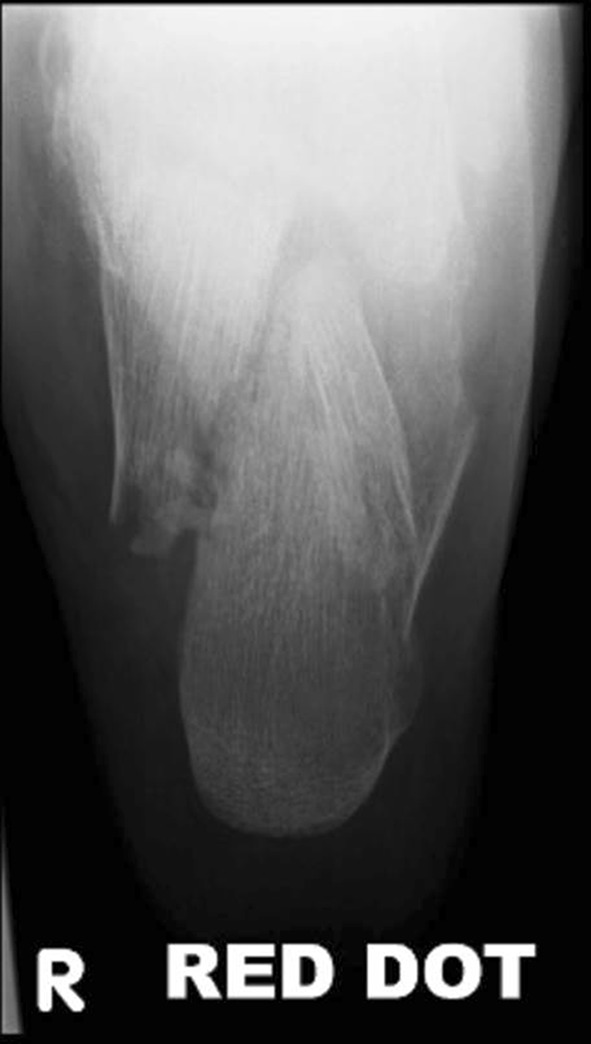
Axial view of fracture in Fig. 1

The two most common are the Essex-Lopresti classification based on the lateral radiograph and the Sanders classification based on coronal CT imaging at the widest part of the posterior facet (Figs. 1 and 2).

**Fig. 3:**
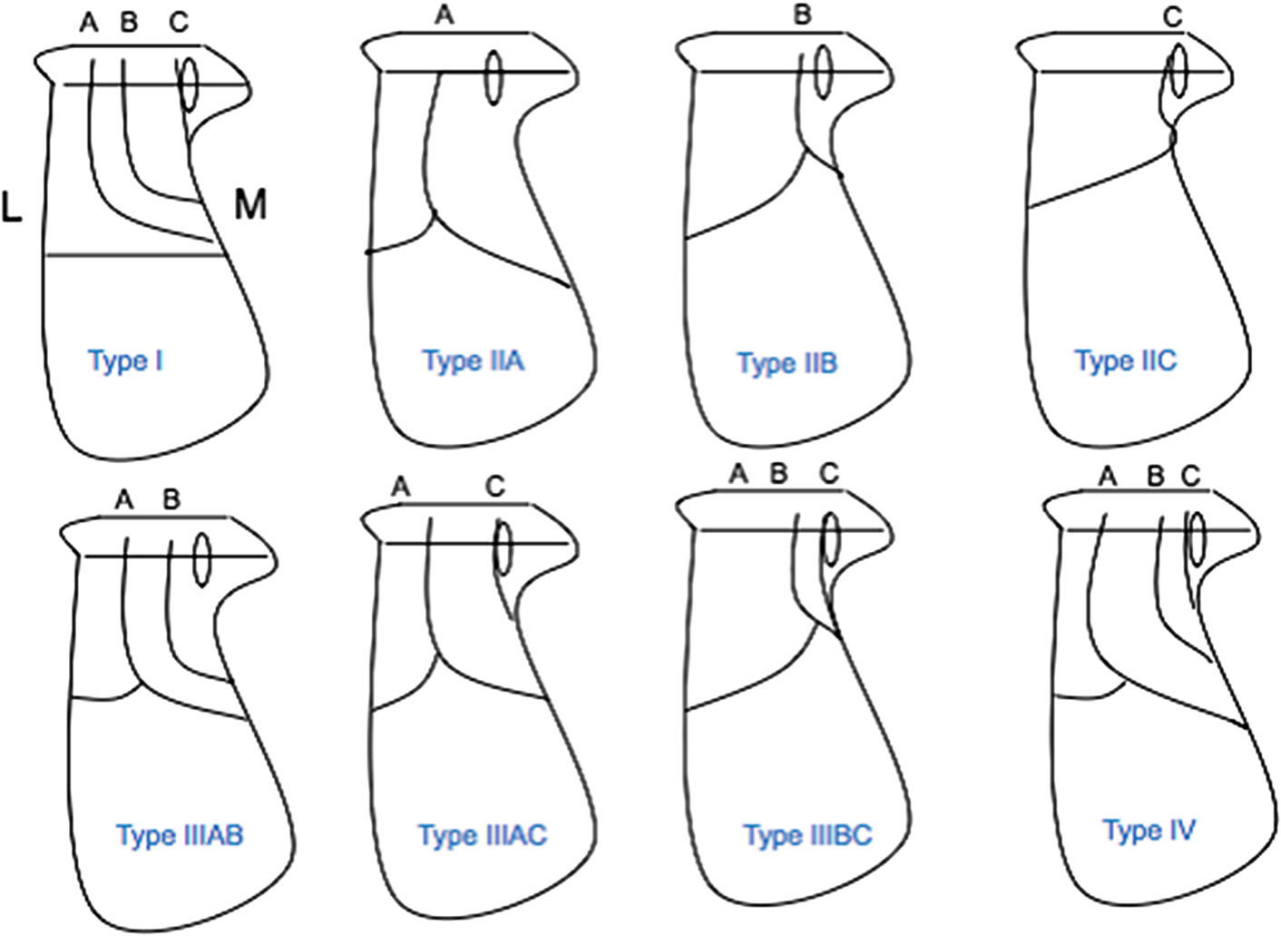
Sanders classification—type I–type IV; based on coronal CT imaging

**Fig. 4:**
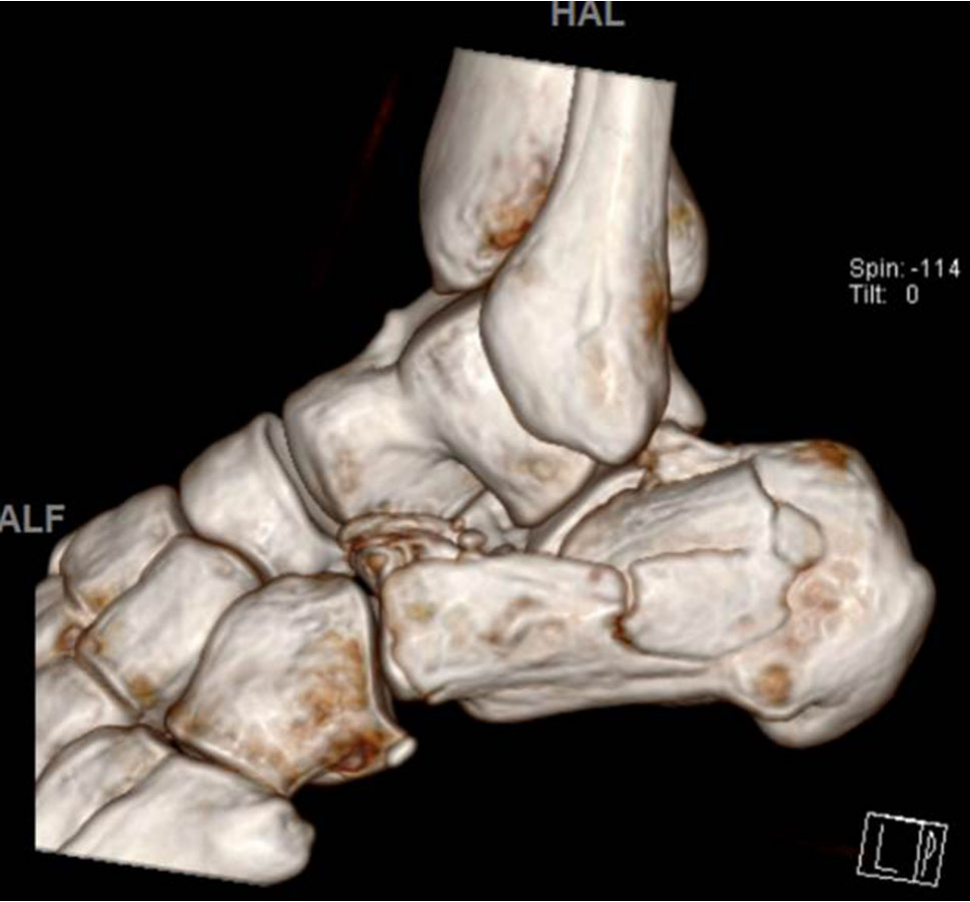
CT reconstruction Sanders II, joint split. Constant fragment medially, lateral articular piece rotated by 90°

**Fig. 5:**
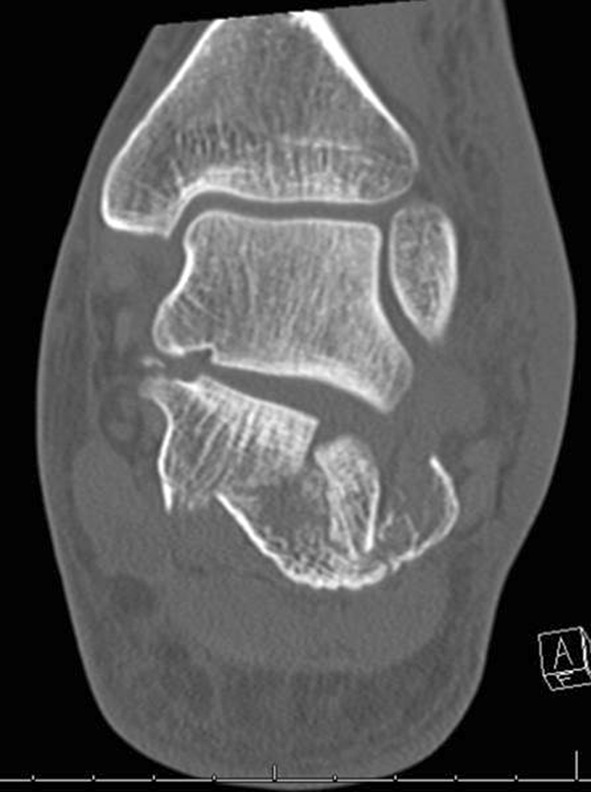
Coronal CT images

**Fig. 6:**
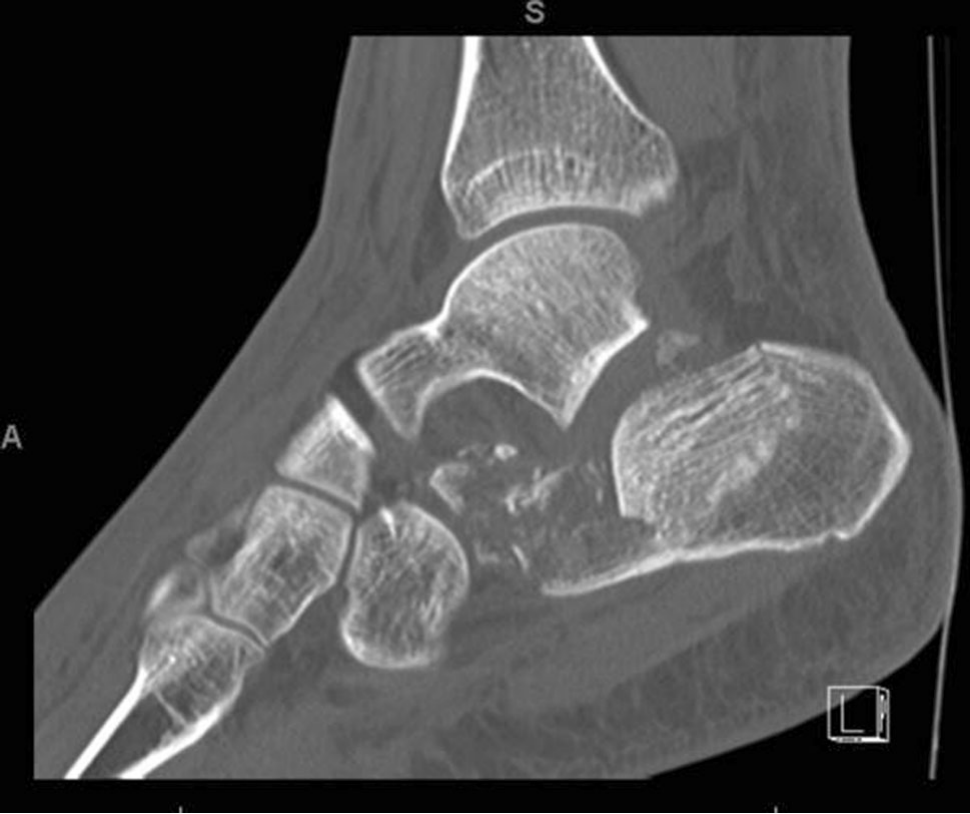
Sagittal CT images

In the Sanders classification, type I fractures are non-displaced or those with less than 2 mm articular displacement, regardless of the number of fracture lines or fragments. Type II fractures are displaced 2-part fractures of the posterior facet with three principal subtypes. Type III fractures are displaced 3-part fractures with an associated central depression with subtypes based on the location of the fracture lines extending into the posterior facet. Type IV fractures are comminuted intra-articular fractures of 4 or more parts, with 3 or more fracture lines extending to the joint and often with significant displacement (Figs. 3, 4, 5 and 6) [10].

Do these classifications systems have clinical relevance and reliability? Sanders found in his study of displaced intra-articular calcaneal fractures that as the number of articular fragments increase, the results and prognosis worsen. Type IV Sanders fractures fared worse after an open reduction and internal fixation [10]. The Sanders classification, although descriptive on fracture line position and prognosis, is limited to the posterior facet of the calcaneum and does not consider the relationship between the three facets of the subtalar joint. The inter-observer reliability reported for the Sanders classification among eight observers reached moderate agreement, but there was poor reproducibility [11]. Other studies have concurred [12].

Open fractures of the calcaneum are severe, potentially limb-threatening injuries that result from high-energy mechanisms. However, there is an increasing incidence of open calcaneal fractures in the elderly from low-energy mechanisms. Many concerns with these injuries include viability of soft tissue, vascular supply, infection, osteomyelitis, non-anatomical reduction and post-traumatic arthritis. A difficult decision is between salvage and amputation. These fractures tend to be the Sanders types III and IV associated with poorer outcomes. In type I, Gustilo–Anderson (GA) open fractures and those with a well-restored subtalar joint and preserved soft tissue have favourable outcomes predictably [13]. Limitations of this study include a small cohort and inclusion of both extra and intra-articular fractures. A consensus for type III GA fractures is to focus on debridement and prompt soft tissue coverage; early internal fixation is avoided to prevent deep infection and osteomyelitis and an eventual amputation [14].

### Assessment

Calcaneal fractures result from high-energy mechanisms of injury. These patients must be assessed and managed per the Advanced Trauma and Life Support (ATLS) protocol which is evolving [15]. There are high thoracic and lumbar vertebral injuries (~ 10%), contralateral calcaneal injuries (~ 10%), as well as tibial plateau and plafond fractures associated commonly. In particular, there is an association with other foot injuries and talar neck fractures (10%) [16]. These are seen usually in high-energy injuries; a recent study of 45 cases showed that this type of ipsilateral combined injury carries a significant morbidity leading to subtalar arthritis and, in open fractures, amputation below the knee [17]. As the population ages, low-energy injuries in osteoporotic bone can lead to complex fractures and dislocations [18, 19]. A past medical history—peripheral vascular disease, previous hindfoot infection and smoking status—contributes to the overall outcome [20]. In the presence of more than one risk factor, there is a cumulative increase in the relative risk of wound problems [21].

### Examination

The greater the force, the greater the degree of fracture displacement and soft tissue disruption. In the higher-energy circumstances of open fractures, there is serious compromise of the viability of soft tissue and concern for neurological and vascular integrity. The soft tissues dictate the outcome for the patient, and a cautious approach to allow for swelling to reduce and soft tissues to settle is recommended before embarking on fixation [21].

### Imaging

**Fig. 7:**
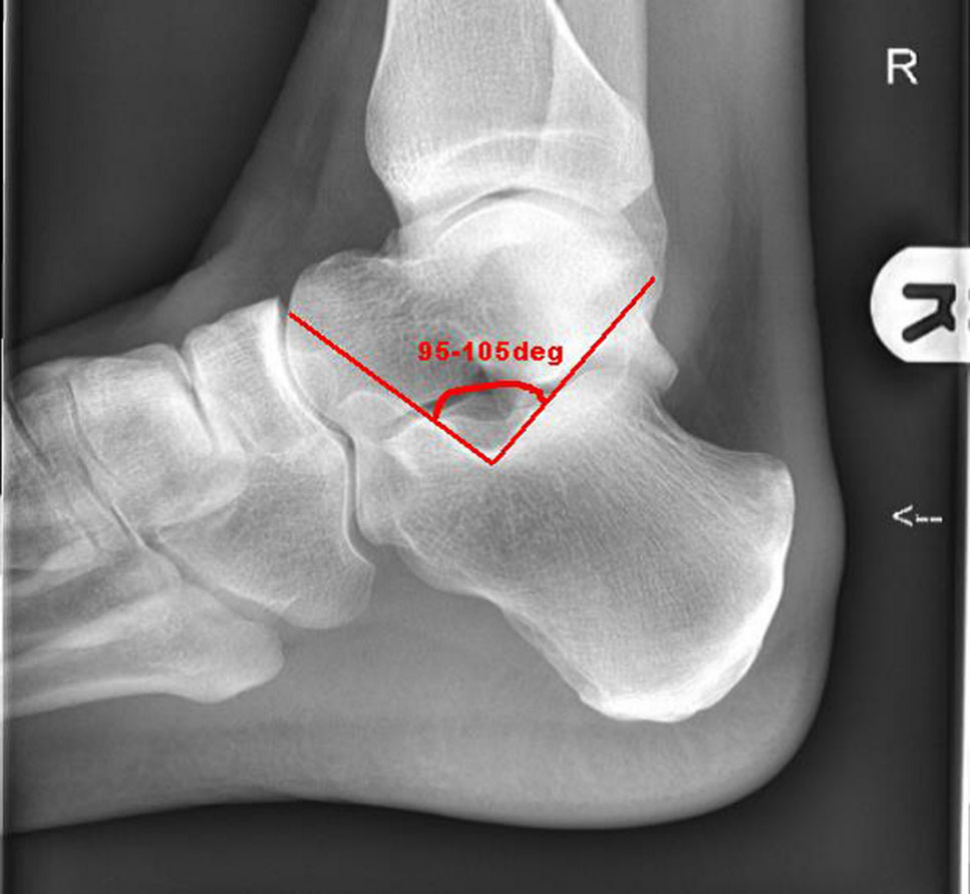
Critical angle of Gissane

**Fig. 8:**
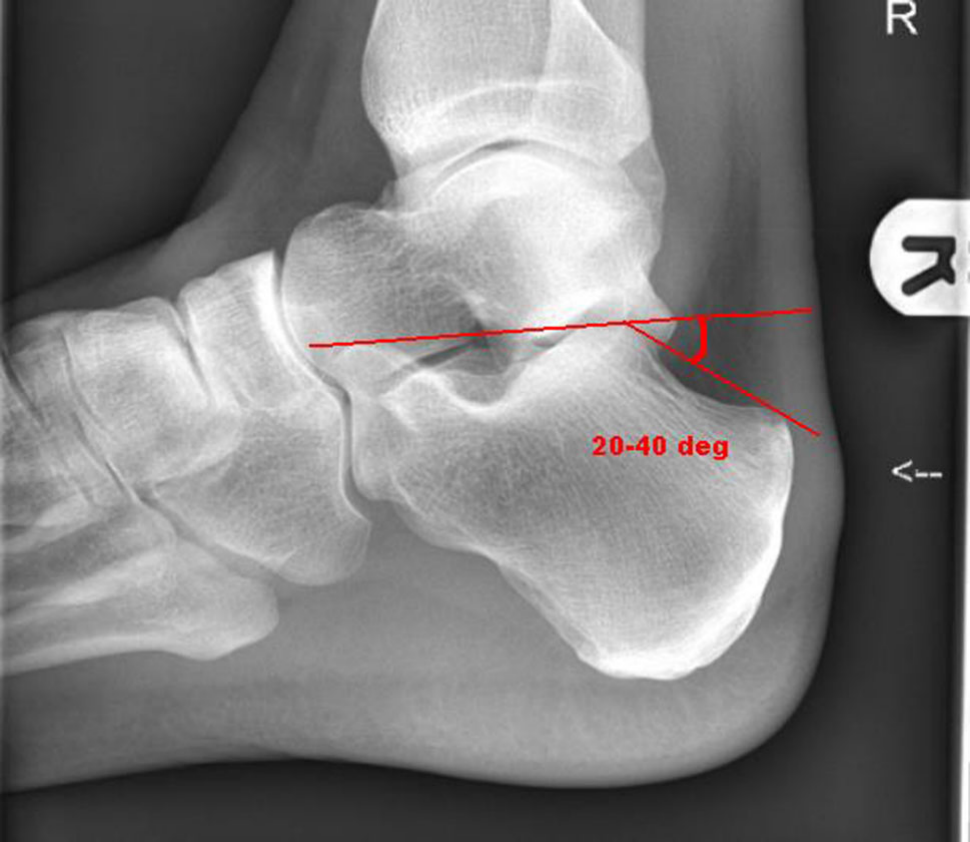
Bohler’s angle

Plain AP and lateral radiographs of the foot and ankle and Harris axial views of the calcaneus are recommended. The two measurements of interest on the lateral radiograph are Bohler’s angle and the critical angle of Gissane. A Bohler angle of less than 20 degrees indicates a collapse of the posterior facet. A reduced angle of Gissane also indicates a collapse of the posterior facet [22] (Figs. 7 and 8). A fine-slice CT scan with multiplanar reconstructions is the gold standard in imaging for calcaneal fractures. This is for classifying the fracture pattern, decision-making and pre-operative planning [23]. An assessment of the loss of normal foot contours can be made from the CT as can the presence of any lateral wall blow out which may lead to fibular impingement—from lateral displacement of the posterolateral fragment.

### Non-operative treatment

Extra-articular calcaneal fractures that do not involve the subtalar joint and are either undisplaced or have minimal displacement can be treated non-operatively. A posterior splint can be applied allowing for ankle and subtalar movement. The patient remains non-weight bearing for a minimum of 4–6 weeks before a graduated increase in weight bearing is permitted.

Of the intra-articular fractures of the calcaneum, Sanders type I fractures may be treated non-operatively. Sanders types II and III fractures are controversial fracture types where surgical management has been appraised critically from outcomes and prognosis; it is reasonable to treat a minimally displaced Sanders II fracture non-operatively [24, 25].

Prognosis varies with the extent and type of intra-articular fracture. Return to work by 4 months (light duty) and back to previous employment at 6 months are reported in one study [24]. Another study comparing operative versus non-operative management over a 6-year period revealed that non-operatively managed patients had good results: all had returned to work; all but one returned to physical activity; and none required a subtalar arthrodesis. By contrast, accurate operative fixation heralded good results as well [25].

There is evidence to support non-operative management in non- and minimally displaced fractures. Those with significant displacement do not show the same results. Some papers, in comparing operative to non-operative management, are at risk of selection bias—the displaced fractures being operatively treated and those undisplaced being treated without surgery [1, 3, 24, 42].

### Operative fixation

Historically most calcaneal fractures were treated non-operatively and, in some, surgical fixation contraindicated [26]. An early surgical technique involved placing a sandbag medially and a pad over the lateral side which, using a hammer, was struck to reduce the lateral wall [27]. There were high rates of malunions and fractures using this method [28]. Operative treatment was avoided until the 1930s for many reasons, including a lack of understanding of the fracture pattern and its natural history, the lack of antimicrobial therapy, satisfactory fixation technology and fluoroscopic imaging.

Modern operative stabilisation is founded on the following steps.

### Surgical approaches

#### Sustentaculum tali fractures

The medial approach gains good access, to the sustentaculum tali, inferior and medial aspect of the calcaneus. This is the approach of choice for open calcaneal fractures as it is where the sustentaculum tali exits through the skin. Due care is needed with the neurovascular structures, particularly the medial calcaneal nerve, and the tibialis posterior tendon. The first interval is developed between the tibialis posterior tendon and flexor hallucis longus tendon (FHL). Deep to FHL lies the medial wall of the calcaneus and the sustentaculum tali [29, 30].

#### Intra-articular fractures: Sanders II, III and IV

The extended lateral approach is the most common approach used to access most displaced intra-articular calcaneal fractures where access to the posterior facet, posterolateral, anterolateral fragment and subtalar joint is required [31, 32]. The blood supply (peroneal and lateral calcaneal artery) to the skin is at risk, and it is important to have a full thickness flap to avoid skin necrosis. This flap should reveal the subtalar joint and the sinus tarsi. The sural nerve is encountered if there is extension of this approach [33, 34].

**Fig. 9:**
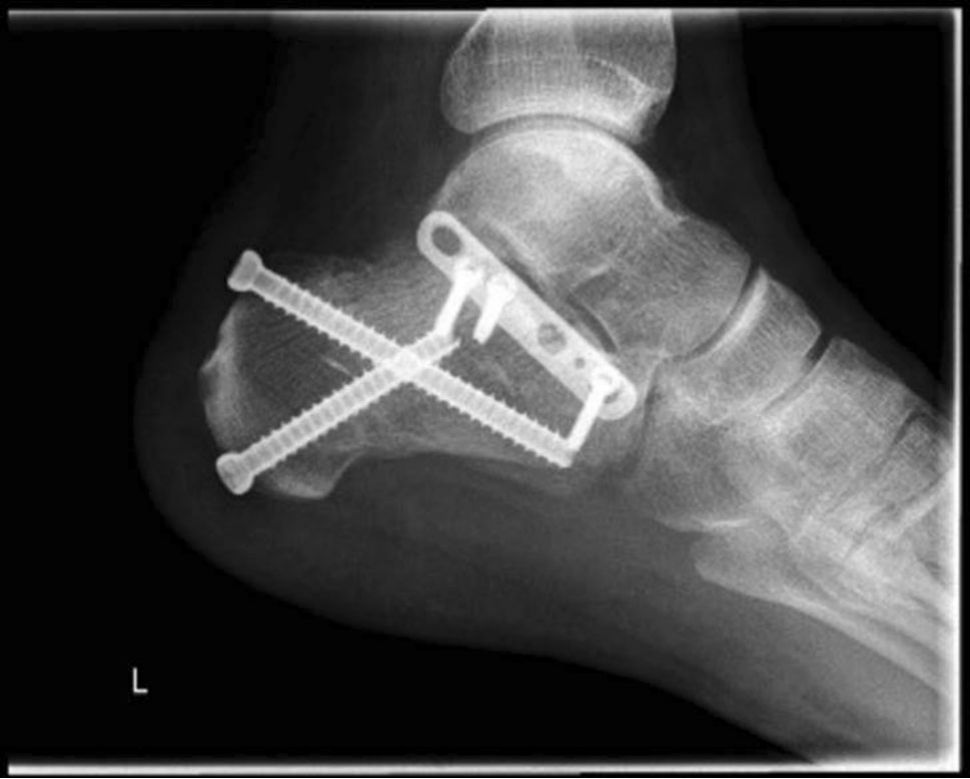
Limited sinus tarsi approach for lag screw fixation and lateral wall plate plus percutaneous screws

The sinus tarsi approach, a limited lateral approach, is tailored approach that allows access to the subtalar and calcaneocuboid joint. One study demonstrated that there were fewer wound complications and better preservation of lateral skin flap blood supply in Sanders II and III fractures, although this study had a small cohort of 13 patients (Fig. 9) [35]. Another review of 271 displaced intra-articular calcaneal fractures found the outcome and complication rates of the sinus tarsi approach were comparable to the established lateral extensile approach, but there was concern in the level of anatomical reduction using this approach [36]. A combined medial and lateral approach has been described for displaced intra-articular fractures which has wound complications [37].

### Timing of surgery

The timing of surgery is important; early surgery (< 3 days from injury) runs the risk of wound breakdown and necrosis. Preparation before surgery includes close monitoring of the skin and use of elevation and ice if appropriate. If, when the ankle is dorsiflexed and everted (and often in the second week post-injury), direct visualisation of the lateral aspect of the calcaneus reveals wrinkling of the skin, then surgery is safe to proceed [38]. Observing for compartment syndrome is important.

### Aims of surgery

The surgical goals of managing calcaneal fractures have remained unchanged: bony union in the absence of infection and early functional return. The priorities in operative fixation include restoration of heel height and correction of heel varus, tuberosity fragment control, subtalar joint reconstruction, reconstruction of the medial and lateral walls and release and protection of the tendons and neurovascular structures.

### Surgical technique

**Fig. 10:**
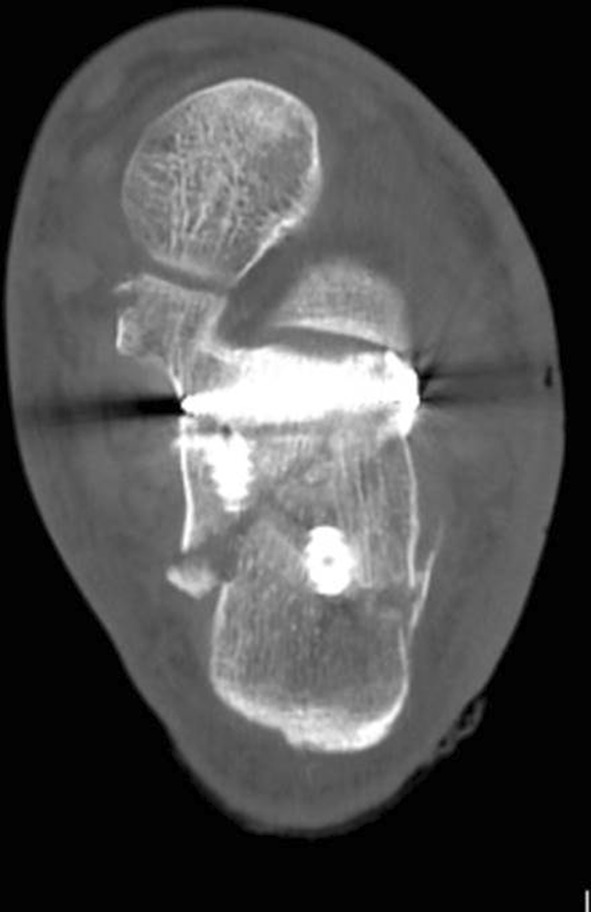
Length and lateral translation of tuberosity restored with articular reduction within 1-mm, axial view CT

**Fig. 11:**
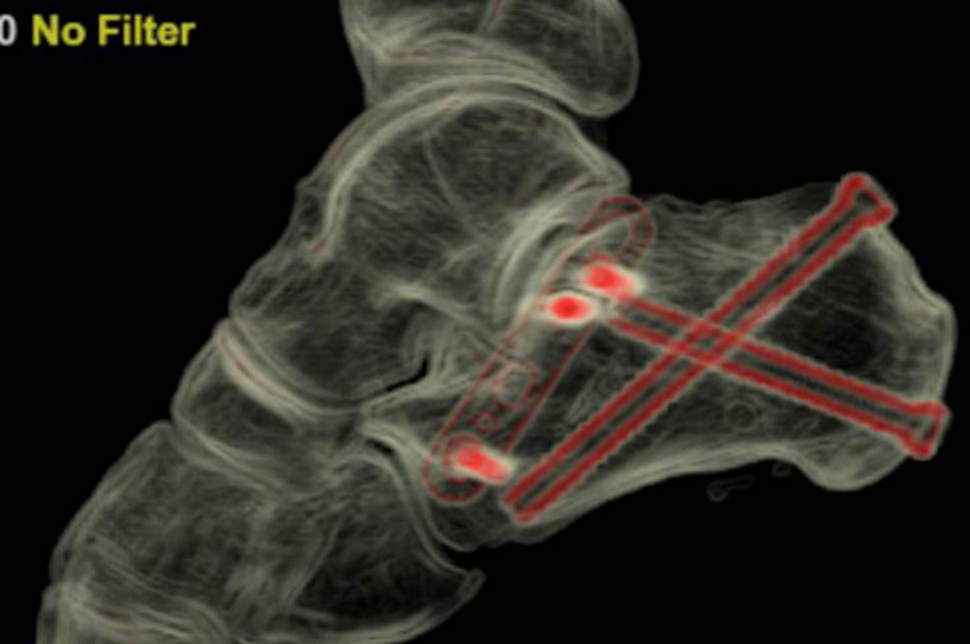
Reconstructed post-operative sagittal view from CT

**Fig. 12:**
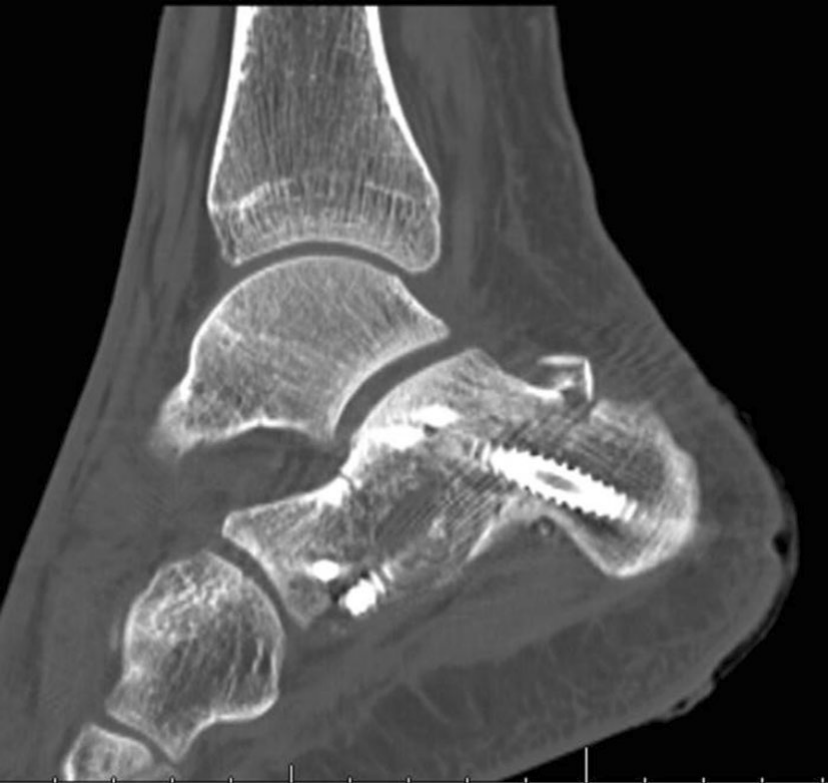
Post-operative sagittal CT views

In the lateral and extended lateral approaches, the lateral wall fragment can be reflected or temporarily removed as needed. Many techniques in operative fixation have used the a Schanz pin to manipulate the tuberosity of the calcaneus in Sanders types II, III and joint depression-type fracture patterns to align it and ensure anatomical reduction [39, 40]. Manipulation of the depressed posterior facet fragment(s) is next, elevating to meet the constant medial sustentacular fragment. Kirschner wires or non-locking screws can be passed from the lateral wall into the sustentaculum bone medially. Bone graft at this stage can sometimes be used to fill in the void created beneath the elevated articular fragments or for comminution. A low-profile lateral calcaneal plate is applied acting as a strut for the posterior tuberosity and posterior facet and, in effect, recreates the lateral wall (Figs. 10, 11 and 12).

In tongue-type fractures, a Schanz pin can be placed lateral to the Achilles tendon and into the superior part of the large tuberosity fragment to manipulate its reduction and to disimpact the articular fragment. A sinus tarsi approach can then be performed to assess the articular surface reduction which is stabilised with a series of wires from anterior to posterior, followed by axial wires from posterolateral to anteromedial into the sustentaculum. Cannulated screws can then be passed over the AP wires to provide compression, followed by axial cannulated screws. The lateral articular fragment can then be elevated to restore the articular surface. A lag screw can then be inserted from the lateral cortex towards the sustentaculum tali capturing the “constant fragment”.

### Plate choice

The purpose of the plate is to provide 3-point fixation across the calcaneal body and to be low in profile so as to avoid skin tension. A one-third tubular plate can be used for displaced simple fracture patterns [41]. In a calcaneal fracture that is comminuted and has poor bone stock, a locking plate may be more appropriate. One study of 28 patients with joint depression-type calcaneal fractures treated with a locking plate reported that 86% of patients had excellent-to-good results based on AOFAS scores, good functional outcomes and restoration of anatomy [42].

A cadaveric study compared a conventional calcaneal plate to a low-profile locking plate on fracture reduction and failure of implant with cyclical loading. The locking plate showed a lower deformation rate and significantly higher load to failure compared with the conventional one [43]. Another group looked at the differences between uniaxial and polyaxial screws in locking plates using calcaneal saw bones and found that during cyclical loading the plate with the polyaxial screws showed less displacement and hence increased stability [44].

### Is the constant fragment really constant?

The sustentaculum fragment, otherwise known as the “constant fragment”, is anatomically bound to the talus by the deltoid ligament and the interosseous ligament. It is described as the medial calcaneal building block to which the remainder of the calcaneus should be built. It has been reported, however, that the “constant fragment” may not be all that constant. In a study of 80 patients with 100 displaced intra-articular fractures, 42 of these fractures demonstrated an increased risk of angulation and translation of the sustentaculum fragment. This was mostly seen in Sanders types III and IV fractures and highlights a potential pitfall to the quality of reduction when constant fragment is referenced [45].

### Outcomes of fixation

Displaced intra-articular fractures remain an area of controversy with regard to operative management. Several small studies have not demonstrated differences in outcomes between operatively and non-operatively managed calcaneus fractures [46]. Some studies have shown that restoring Bohler’s angle is key in achieving a good functional outcome [25].

Sanders, in a review of 148 patients with displaced intra-articular fractures over a 4-year period, found that 79 had Sanders type II fractures of which 86% were anatomically reduced. Of the 30 Sanders type III fractures, 60% had anatomic reduction. In the Sanders type IV group, none were anatomically reduced. This group, as expected, had the highest complication rate in wound problems, sural nerve injury, peroneal tendon injury and infection. Sanders type IV fractures continue to be the most challenging technically, and it was recommended that subtalar arthrodesis be the treatment of choice for these patients [10].

Buckley conducted a multicentre trial including 424 patients comparing non-operative treatment of Sanders types II and III intra-articular calcaneal fractures with operative. He demonstrated that non-operative treatment was appropriate for certain patients with a potential need for late subtalar arthrodesis, and there was no difference in the clinical outcomes from operative treatment. It, too, failed to show any significant difference between the radiological outcomes after operative fixation when compared with non-operative. The study did highlight the importance of anatomic reduction in surgery producing better long-term outcomes. Despite this study being a multicentre trial, 73% of the fractures were operated on by one surgeon. This raises an element of operator bias [47].

A large multicentre trial, the UK Heel trial, compared operative management of closed intra-articular calcaneal fractures with non-operative treatment. Results showed no difference in outcome between the two groups based on functional outcome scores. There was a high rate of surgical site infections and reoperations [48]. The exclusions in this study included bilateral fractures, open fractures, those with fibula impingement and extra-articular fractures. The follow-up was to 2 years; this period may have been insufficient to detect post-traumatic arthrosis.

Despite the evidence above, the general opinion from the literature is Sanders types II and III fracture patterns with good or excellent functional outcomes from surgery. The associated wound complications, osteomyelitis, non- or malunion have been reduced, possibly from advances in surgical technique, implants and antimicrobial therapy. But in the absence of compelling evidence to support this, it is likely that these fractures will be treated conservatively to avoid complications.

### Minimally invasive surgery

Complication rates from open reduction and internal fixation of displaced intra-articular fractures are high. Minimally invasive surgery (MIS) offers an alternative approach, which minimises the dissection and subsequently risk of wound dehiscence, infection, arthritis [49]. The technique itself is variable from the different studies published, and some describe a dual technique with a semi-open reduction followed by internal fixation with a minimally invasive technique [50].

The advantage of MIS is early surgical intervention, at 2 or 3 days post-injury. A Steinman pin in the postero-inferior aspect of the calcaneal tuberosity is used to aid traction and reduction. Thru a second percutaneous incision, the depressed or displaced lateral fragment is elevated and reduced. Two K-wires are then placed to secure the reduction in the tuberosity to the sustentacular fragment [49].

There have been several case series with positive results, supporting minimally invasive surgery (MIS) [51–53]. Comparative studies have looked at the use of percutaneous reduction and screw fixation as compared with open reduction and internal fixation; these small studies have shown lower infection rates in fractures treated percutaneously although the quality of reduction and sample sizes were limiting factors [54].

There appears to be little difference between the sinus tarsi approach discussed earlier and the MIS lateral approach. However, the MIS approach is not just limited to the lateral side; Carr describes a modified medial approach which protects the neurovascular bundle and allows application of a small antiglide plate [55]. MIS has benefits of a reduced risk of complications but requires an experienced surgeon.

### Use of subtalar arthroscopy in calcaneal fracture fixation

Intra-operative use of fluoroscopy to assess the reduction in intra-articular calcaneal fractures is standard. Subtalar arthroscopic-assisted fixation has been described as a means of assessing articular reduction intra-operatively [56]. A study has demonstrated how arthroscopy can assess the quality of reduction but needs a surgeon experienced with the arthroscopic technique [57, 58]; the argument is that if reduction is assessed directly, arthroscopic assessment prolongs anaesthetic and surgical time.

### Arthrodesis

There are some patients where a subtalar arthrodesis is the recommended form of treatment, usually the grossly comminuted intra-articular fractures of the posterior facet (Sanders type IV). The timing of arthrodesis is controversial. Primary arthrodesis of the subtalar joint is an option in the treatment for a subset of calcaneal fractures, usually the most severely comminuted intra-articular fractures. Buckley demonstrated that Sanders type IV fractures have poor outcomes with either operative or non-operative treatment [47]. Several proposed reasons are: in comminuted fractures anatomical reconstruction of the posterior articular facet is not usually possible [10]; in high-energy injuries irreversible cartilage damage occurs at the time of fracture [59]; post-traumatic arthrosis is reported as high as 71% and the need for secondary fusion is 5.5 times higher in this subgroup [60].

Favourable outcomes following primary fusion have been reported by several authors. A systematic review by Schepers et al. in 2012 summarised the published literature from 1990 to 2010 [61]. A total of eight case series were included describing the outcome of 128 fractures in 120 patients: an average modified AOFAS score of 77.4 (0–94) was reported; union rates ranged from 90 to 100%; return to work was 75–100%; and wound complications and infection featured in 19.4% with three amputations.

The surgical technique for primary arthrodesis varies. Classically, a posterolateral approach to the subtalar joint is used, but recent awareness for reconstruction of normal calcaneal height and alignment has led to a trend to open reconstruction and arthrodesis [62]. Primary arthrodesis is an option for highly comminuted intra-articular calcaneal fractures. There is, as yet, no published evidence to support one fusion technique over another or the superiority of fusion over non-operative treatment or ORIF.

## Discussion

The optimum management of calcaneal fractures remains controversial; the role for surgery is not established due, in part, to the poor quality of existing published data. Most studies have weaknesses in design, power and control of bias to answer the question as to which patients may benefit from surgery. The more recent and better designed trials have failed to demonstrate the benefits of operative treatment. The identification of a cohort of patients that will benefit from surgery remains elusive, suggesting that any real gains from surgery may be marginal. Added to this is clear evidence of significant complications associated with surgery; the argument for operative treatment becomes difficult.

There is no evidence that operative treatment is suitable for all fracture types. Fracture severity, as classified by Sanders, is used to direct treatment. Undisplaced (type I) fractures should be treated non-operatively. Displaced (types II and III) unilateral calcaneal fractures with intact soft tissue envelopes and no “gross displacement” or “fibular impingement” are best treated non-operatively. This recommendation bears particular validity where surgeons are unfamiliar or unpractised in open reduction and internal fixation of calcaneal fractures. Until then, evidence is needed to support a reappraisal of role of surgery in these injuries, especially if outcomes are improved through advances in surgical approach, intra-operative imaging, fixation technique, and familiarity in the hands of “experts”. If so, the balance of risk versus benefit may be re-addressed in this cohort of patients.

Highly comminuted (type IV) fractures have poor results from either non-operative treatment or ORIF. There is potentially a role for primary reconstruction through arthrodesis, and a Canadian multicentre RCT currently in progress may clarify this. (http://clinicaltrials.gov/show/NCT00679393).

Patient-related factors, as distinct from the fracture characteristics, influence operative outcome significantly. Smoking, peripheral vascular disease and diabetes are associated with wound complications and infection. Worse outcomes are reported in men, those over 50s and patients claiming workers’ compensation. There are some fractures for which surgery is the preferred option intuitively. Open fractures, grossly displaced fractures with severe impingement of soft tissues and those with impending skin breakdown (tongue-type fractures) are treated operatively. Direct evidence in support is absent, but this approach is made on the basis of the likely prognosis if left untreated; a RCT to provide an evidence-based decision may generate issues over equipoise in anticipated risks and benefits such as to be impracticable. Until then, it is likely surgeons will continue to treat these severe injuries operatively.

If benefits to operative treatment of calcaneal fractures are to be established, the focus of future work will be to identify that combination of patient, fracture and surgical techniques.

## Conclusion

The spectrum of injuries to the calcaneus ranges from undisplaced extra-articular fractures to open, comminuted intra-articular fractures. Many challenges are faced in managing these fractures including restoring the articular joint surface, the posterior facet and maintaining a good non-infected soft tissue envelope over the fracture site, with a normal foot shape and profile. Recent studies have again shown no differences in outcome between non-operative and operative management. Good functional outcomes with concomitant lower complication rates are being seen with improved surgical techniques; however, no consensus or evidence remains for a gold-standard treatment for these injuries.
